# CD8 T Cell Memory Recall Is Enhanced by Novel Direct Interactions with CD4 T Cells Enabled by MHC Class II Transferred from APCs

**DOI:** 10.1371/journal.pone.0056999

**Published:** 2013-02-18

**Authors:** Pablo A. Romagnoli, Mary F. Premenko-Lanier, Gilbert D. Loria, John D. Altman

**Affiliations:** 1 Emory Vaccine Center and Department of Microbiology and Immunology, Yerkes National Primate Research Center and Emory University School of Medicine, Atlanta, Georgia, United States; 2 Division of Experimental Medicine, University of California San Francisco and San Francisco General Hospital, San Francisco, California, United States; MRC National Institute for Medical Research, United Kingdom

## Abstract

Protection against many intracellular pathogens is provided by CD8 T cells, which are thought to need CD4 T cell help to develop into effective memory CD8 T cells. Because murine CD8 T cells do not transcribe MHC class II (MHC-II) genes, several models have proposed antigen presenting cells (APCs) as intermediaries required for CD4 T cells to deliver their help to CD8 T cells. Here, we demonstrate the presence of MHC-II molecules on activated murine CD8 T cells in vitro as well as in vivo. These MHC-II molecules are acquired via trogocytosis by CD8 T cells from their activating APCs, particularly CD11c positive dendritic cells (DCs). Transferred MHC-II molecules on activated murine CD8 T cells were functionally competent in stimulating specific indicator CD4 T cells. CD8 T cells that were “helped” in vitro and subsequently allowed to rest in vivo showed enhanced recall responses upon challenge compared to “helpless” CD8 T cells; in contrast, no differences were seen upon immediate challenge. These data indicate that direct CD8∶CD4 T cell interactions may significantly contribute to help for CD8 T cells. Furthermore, this mechanism may enable CD8 T cells to communicate with different subsets of interacting CD4 T cells that could modulate immune responses.

## Introduction

Immunological memory to intracellular pathogens is mediated in many cases by CD8 T cells [1]. In consequence, defining the precise mechanism by which memory CD8 T cells are generated is essential to improve the quality and effectiveness of vaccines for such pathogens.

CD8 T cells must receive more than one signal of activation to become fully functional [2]. Signal 1 is provided when the T cell receptor (TCR) on CD8 T cells recognizes its cognate peptide presented in the groove of MHC class I molecules on antigen presenting cells (APCs) [3], usually a dendritic cell (DC) [4]. Signal 2 is provided by costimulatory molecules, typically members of the B7 family [5] or the TNF family [6] or chemokines [7], also expressed on DCs activated by inflammatory pathogen-associated molecular patterns (PAMPs) [8]. Lastly, a third signal given by cytokines present in the surrounding inflammatory milieu [9] completes the activation phase of a nascent CD8 T cell response.

In addition to the signals mentioned above, to become functional long term memory cells, CD8 T cells require additional signals from CD4 T cells [10]. It has been reported that when CD4 T cells are depleted or absent, memory recall responses by CD8 T cells are impaired [11], [12], [13], [14]. However, whereas some of the signals involved in the CD4 T cell help have been identified [15], [16], [17], [18], [19], [20], [21], the precise mechanism by which CD4 T cells provide help for CD8 T cells remains poorly understood.

A major conceptual roadblock to understanding how CD4 T cells provide help to CD8 T cells is that while all other immune cells that require help – e.g B cells and macrophages – transcribe and translate MHC-II, murine CD8 T cells mostly do not, an effect that has been tied to the hypermethylation in promoter III of the transcription factor MHC-II Trans Activator (CIITA) [22]. In contrast, it has been shown that human activated CD8 T cells express MHC-II [23], though the immunological significance of this observation has never been satisfactorily addressed.

While the data reporting the failure of murine CD8 T cells to transcribe MHC-II appears to be very solid, scattered reports over the course of 30 years have described MHC-II on mouse T cells [24], [25], [26], [27] and have suggested that the cells may acquire MHC-II from other cell types by a membrane transfer mechanism recently termed trogocytosis [28], [29], [30], [31], [32]. In this report we further verify that activated CD8 T cells become MHC-II positive during the early stages of antigen recognition and that these MHC-II molecules are derived from APCs, principally CD11c+ DCs. We also show that the transfer of MHC-II together with their peptide ligands endows CD8 T cells with the ability to interact directly with helper CD4 T cells which in turn deliver signals that confer to the activated CD8 T cell the ability to become a long term memory cell.

## Results

### MHC-II is present on activated murine CD8 T cells in vitro as well as in vivo

**Figure 1:**
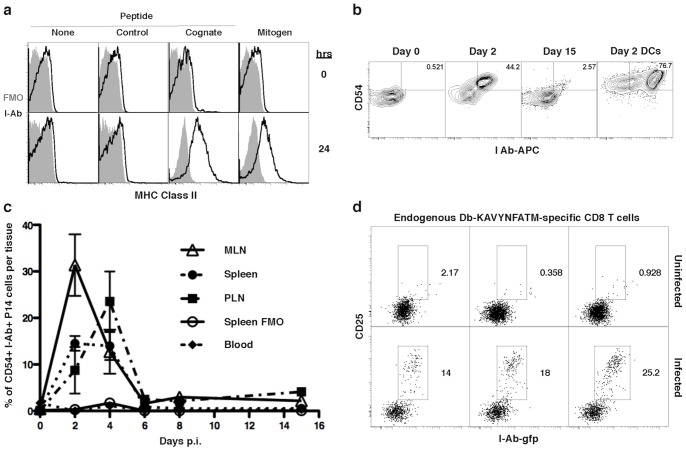
MHC-II is present on activated CD8 T cells in vitro as well as in vivo. **a**. MHC- II staining on Tg CD8 T cells (P14 cells) shown at times 0 and 24 hrs after in vitro incubation with WT flt3L-DCs pulsed with vehicle, control peptide (ova257-64), cognate peptide (gp33-41) or mitogen (Con A). Solid histograms: fluorescence minus one (FMO) control. Empty histograms: I-A^b^–FITC stained. Events were gated on CD3+CD8+ singlets. Histograms are representative of at least three independent experiments. **b.** CD54 and MHC-II (I-A^b^) staining on P14 cells in MLN at days 0, 2 and 15 after infection with 2×10^5^ p.f.u. of LCMV Arm i.p. Plots are representative of triplicates. Events gated on live CD19−Thy1.1+CD8+ singlets. Day 2 DCs in MLN are gated on live CD11c+ singlets. **c.** Percentage of CD54+ and I-Ab+ P14 cells in MLN, Spleen, PLN, Spleen FMO and Blood at different times (0, 2, 4, 6, 8 and 15 days) after infection with 2×10^5^ p.f.u. of LCMV Arm i.p. Each point is represented by the mean and SEM of triplicates. **d.** MHC-II (I-A^b^-gfp) vs CD25 staining on activated D(b)/LCMV.gp33-41 (KAVYNFATM) tetramer enriched CD8 T cells 2.5 days p.i. with 2×10^6^ of LCMV Arm i.v. One graph per mouse. Events gated on live CD19−CD11b−CD4−CD8+ KAVYNFATM-tetramer+ singlets. Plots are representative of triplicates from one of two independent experiments.

Although it is known that murine CD8 T cells can not transcribe MHC-II genes [22], the presence of MHC-II protein on activated CD8 T cells has been described after interaction with APCs [29]. To verify this, we incubated magnetically sorted (purity ∼85%, data not shown) P14 TCR transgenic CD8 T cells (P14 cells) with flt3L in vivo expanded CD11c-enriched DCs (flt3L-DCs) pulsed with one of the following: vehicle, control peptide (Ova257-264), the mitogen Con A, or the stimulatory cognate peptide (LCMV.gp33-41). We found that MHC-II was displayed only on the surface of CD8 T cells activated with either their cognate peptide or with Con A (Fig. 1a).

To determine if a similar event occurs in vivo, P14 cells (1×10^6^) were adoptively transferred into WT mice that were infected one day later with 2×10^5^ p.f.u. of LCMV Arm i.p.. At two days post-infection (p.i.) in the draining mesenteric lymph node (MLN), MHC-II was detected on transferred cells that had upregulated CD54 (ICAM-1), a marker of activation, but not on the approximately 50% of transferred cells which remained CD54^lo^ (Fig. 1b); detection of MHC-II on the CD54^hi^ cells was transient and disappeared by 6 days post-infection (Fig. 1c, Fig. S1). Similar results were found in the spleen and peripheral lymph nodes (PLN) (Fig. 1c, Fig. S1), but with slightly different kinetics. The level of MHC-II detected on activated CD8 T cells is intermediate in comparison to the level of MHC-II displayed by DCs in the same tissue at the same time (Fig. 1b). As expected, MHC-II was not detected on bystander CD8 T cells in vitro or in vivo (Fig. S2). Together, these results definitively show the presence of MHC-II on recently activated CD8 T cells responding to an infection in vivo.

To investigate if non-transgenic CD8 T cells display MHC-II when responding to an infection, WT mice were infected with 2×10^6^ p.f.u. of LCMV Arm i.v.. Spleens, PLNs and MLNs were collected and LCMV-specific CD8 T cells were enriched magnetically using D(b)/LCMV.gp33-41(KAVYNFATM) tetramers. At 2.5 days p.i., enriched cells that expressed CD25 –indicating in vivo activation– also had detectable MHC-II, which was not found on CD25^neg^ cells (Fig. 1d). Even though the percentage of LCMV-specific CD8 T cells activated in vivo that display MHC-II differs slightly from the one observed on P14 cells activated both in vitro and in vivo (Fig. 1), we confirmed their activation level by the increase in complexity that correlates with blasting of activated cells (Fig. S3a). As expected, bystander Kb/Ovalbumin.ova257-64 (SIINFEKL) tetramer enriched CD8 T cells did not show any MHC-II staining since they all remained naïve (CD25^neg^) and were not responding to infection (Fig. S3b). Taken together, both in vitro and in vivo results show that recently activated CD8 T cells display MHC-II on their surface.

### MHC-II is transiently present on responding transgenic CD8 T cells after infection

**Figure 2:**
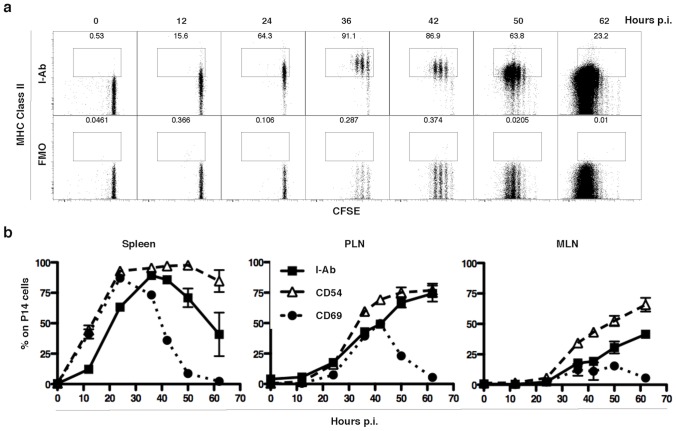
MHC-II is transiently present on responding transgenic CD8 T after infection. **a.** MHC-II staining vs CFSE dilution on P14 cells from spleen at different times (0, 12, 24, 36, 42, 50 and 62 hrs) after infection with 2×10^6^ p.f.u. LCMV Arm i.v.. Plots are representative of duplicates. First row: I-A^b^-APC staining. Second row: FMO control. Events gated on live CD19−CD11c−Thy1.1+CD8+ singlets. **b.** MHC-II (I-Ab), CD54 and CD69 expression on CD8 T cells in spleen, PLN and MLN at different times (0, 12, 24, 36, 42, 50 and 62 hrs) after infection with 2×10^6^ p.f.u. LCMV Arm i.v.. Each point is represented by the mean and SEM.

To determine the kinetics of MHC-II presence on CD8 T cells in vivo, CFSE-labeled P14 cells (1×10^6^) were adoptively transferred into WT mice, and were infected one day later with 2×10^6^ p.f.u. of LCMV Arm i.v.. As shown in Fig. 2a, transferred transgenic CD8 T cells in the spleen start to display detectable MHC-II protein as soon as 12 hours p.i., even before they begin dividing. Division commences between 24 and 36 hours, with the fraction of cells with detectable MHC-II peaking between 36–42 hours post-infection and decreasing through 62 hrs.

We compared the presence of MHC-II and two markers of T cell activation (CD69 and CD54) on transferred CD8 T cells in spleen, PLN and MLN as a function of time after intravenous infection with LCMV Arm (Fig. 2b). All markers of activation on transferred CD8 T cells, including MHC-II, appeared faster in the spleen than in PLN and MLN. MHC-II on transferred P14 T cells continued to rise in PLN and MLN at 50 and 62 hours p.i. when it was already falling in the spleen. Although expression of MHC-II mRNA can be detected by RT-PCR on activated CD8 T cells following acute LCMV infection (Fig. S4a), no substantial presence of MHC-II was detected by flow cytometry at 36 hours post-infection when CD8 T cells were activated in MHC-II knockout mice (CIIKO, Fig. S4b). However, we cannot completely rule out a very low level of expression of MHC-II by the T cells themselves, since WT CD8 T cells stimulated with CIIKO DCs had a very small frequency of cells with detectable MHC-II compared to CIIKO T cells stimulated with CIIKO DCs (Fig. S4c). These results would suggest that even though transcription and translation of MHC-II genes might occur in CD8 T cells, it can not account for the total amount of MHC-II displayed upon activation in response to viral infection.

### MHC-II is transferred onto CD8 T cells from APCs

**Figure 3:**
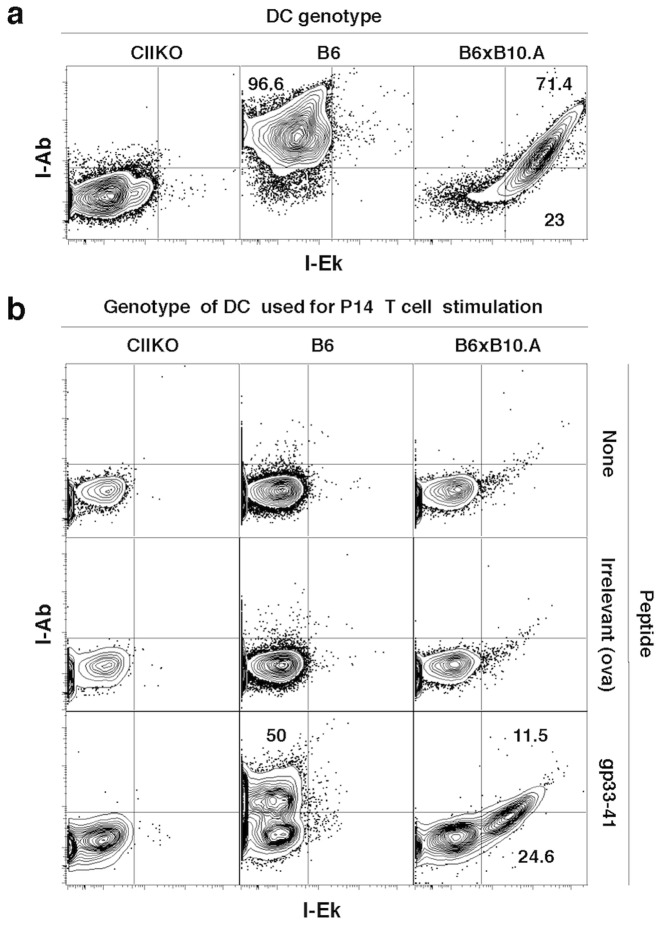
MHC-II is transferred onto CD8 T cells from APCs. **a.** I-A^b^ and I-E^k^ staining on magnetically enriched Flt3L-DCs from CIIKO, B6 and B6×B10.A mice. Events were gated on CD11c+ singlets. **b.** I-A^b^ vs I-E^k^ staining on activated P14 cells after 24 hrs of in vitro culture without peptide, with an irrelevant peptide (ova257-64) or cognate peptide (gp33-41). Events gated on live CD11c-Thy1.1+CD8+ singlets. Plots are representative from one of two independent experiments.

Because mouse CD8 T cells can not express noticeable levels of MHC-II [22], we decided to test the idea that CD8 T cells could acquire these MHC-II molecules by trogocytosis. CD8 T cells were activated in vitro in the presence of flt3L- DCs from either CIIKO, B6 or F1 B6×B10.A mice. As shown in Fig. 3a, CIIKO DCs lacked MHC-II expression, B6 DCs expressed their strain specific MHC-II (I-Ab) and the F1 hybrid DCs expressed both MHC-II molecules derived from its breeder strains (I-Ab and I-Ek).

P14 cells cultured with DCs pulsed with their cognate peptide but not with an irrelevant peptide (ova257-264) or without peptide, displayed the MHC-II that is present on the interacting DCs (Fig. 3b). Since P14 cells have a B6 background and do not have the H-2^k^ alleles, this indicates that the I-E^k^ observed on the activated P14 cells is derived from the F1 APCs. To further test for the source of the MHC-II observed on in vitro activated P14 cells, we performed an experiment in which the MHC-II locus was knocked out (CIIKO) on the T cells, the APCs, or both. Maximal detection of MHC-II on activated P14 CD8 T cells was observed only on T cells cultured with WT DCs, again suggesting that most of the MHC-II observed on the T cells was derived from APCs (Fig. S4c).

To determine what type of APC was best able to transfer MHC-II molecules, we isolated three major populations of APCs from WT mice using positive magnetic enrichment, B220+ containing mostly B cells, CD11b+ containing myeloid DCs, macrophages, monocytes and granulocytes and, CD11c+ containing lymphoid and myeloid DCs (Fig. S5a). Even though all the enriched populations possessed high amounts of MHC-II (Fig. S5b), CD11c+ enriched population transferred the most MHC-II onto CD8 T cells upon in vitro activation compared to B220+ and CD11b+ magnetically enriched populations (Fig. S5c and d). These results further support the fact that MHC-II is transferred from APCs onto activated CD8 T cells and that the APC subset that transfers most effectively are CD11c+ DCs.

### MHC-II on activated CD8 T cells mediate direct stimulation of experienced CD4 T cells

**Figure 4:**
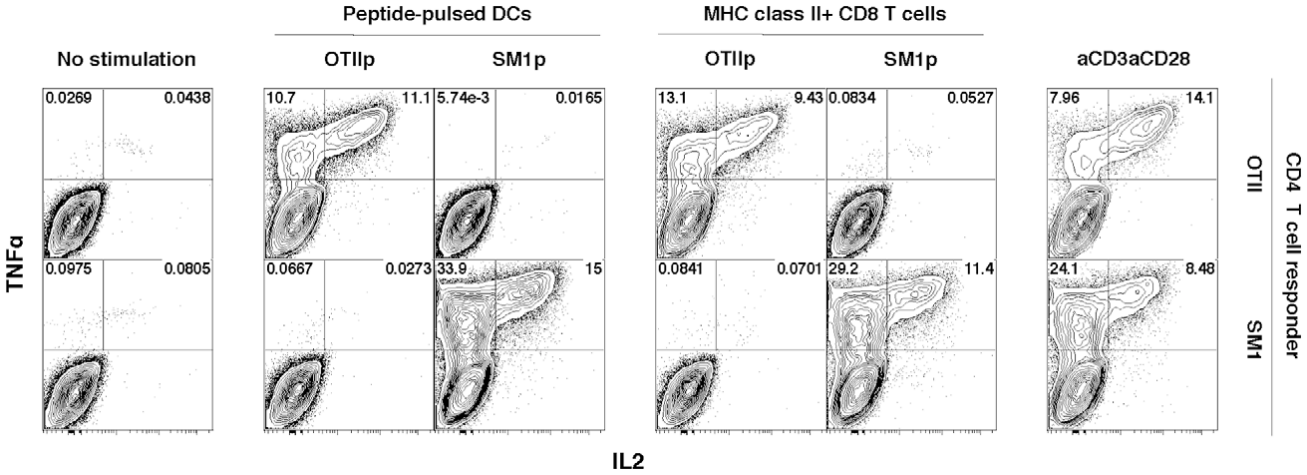
MHC-II on activated CD8 T cells mediate direct stimulation of experienced CD4 T cells. TNFα vs IL2 expression by ICS using flow cytometry on experienced CD4 T cells generated by priming and differentiation with IL2, IL7 and IL-15, stimulated (OTII, upper row; SM1, lower row) by no peptide, DCs or activated Tg CD8 T cells, loaded with either ova353-369 (OTIIp) or FliC427-441 (SM1p) peptide. Events gated on live CD8−CD4+ singlets.

LCMV-specific P14 CD8 T cells were activated as described in Fig. 1a with cognate peptide in addition to MHC-II peptides, ova323-339 for OTIIs (OTIIp) or FliC427-441 for SM1s (SM1p). After activation, CD8 T cells were isolated magnetically and tested for their ability to stimulate in vitro primed OTII or SM1 CD4 T cells (see Material and Methods). As shown in Fig. 4, CD8 T cells presenting MHC-II molecules, obtained from DCs that had been pulsed with MHC-II restricted cognate but not with control peptides, were able to stimulate indicator transgenic CD4 T cells as measured by intracellular staining for TNFα and IL2. In these assays, the antigen presenting CD8 T cells were as potent as peptide-pulsed CD11c+ DCs. Intracellular cytokines were not detected when the CD4 T cells were incubated in the absence of antigen presenting CD8 T cells or DCs, while strong responses were seen when CD4 T cells were stimulated with the combination of anti-CD3 and anti-CD28. These results demonstrate MHC-II/peptide complexes obtained by CD8 T cells from DCs retain full capacity to stimulate CD4 T cells.

To exclude the possibility that the above results were due to DCs contaminating the purified activated CD8 T cells population, two approaches were used. In the first, prior to purification, the CD8 T cell population was spiked with DCs that had been pulsed with the MHC-II restricted peptide that is recognized by the indicator CD4 T cells. In these experiments, the level of contaminating DCs within the purified MHC-II+ CD8 T cells population is below the limit of detection of the flow cytometry assay for CD11c+ cells (Fig. S6a) and insufficient to provide significant stimulation of the indicator CD4 T cells (Fig. S6b). In a second approach, we determined how many DCs contaminating either primed or naïve CD8 T cells are required to provide significant stimulation to indicator CD4 T cells. As shown in Fig. S6c, significant CD4 T cells responses were not seen until the ratio of DCs to CD8 T cells exceeded 1∶40.

### In vitro interactions between MHC-II+ activated CD8 T cells and experienced CD4 T cells improve the in vivo recall response of the CD8 T cells

To test the hypothesis that MHC-II+ CD8 T cells can receive help from CD4 T cells via direct T∶T interactions, we developed a model in lines with a reported model studying memory CD8 T cell differentiation [33] in which we tested the in vivo recall responses of in vitro “helped” CD8 T cells. Prior to transfer into naïve congenic Thy1 mismatched hosts, naïve P14 CD8 T cells were primed on DCs pulsed with both the cognate MHC class I restricted peptide (LCMV.gp33-41) and an MHC-II restricted peptide (either LCMV.gp61-80 or control ova peptide), purified, cultured with in vitro primed SMARTA CD4 T cells, and re-purified. After transfer, recipients were split into two groups. One group was immediately challenged with a recombinant *Listeria monocytogenes* strain expressing the peptide recognized by the CD8 T cells but not the CD4 T cells (rLm.gp33), while the other group was rested for 30 days prior to challenge with the same pathogen. Mice were sacrificed 4 days after challenge and their spleens were analyzed for numbers of Thy1.1+ CD8 T cells (P14).

**Figure 5:**
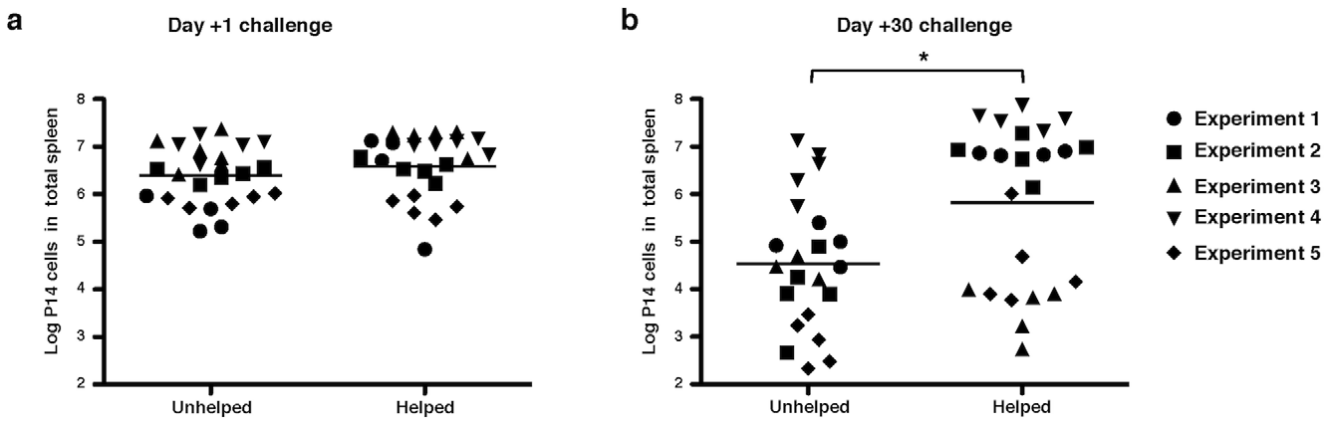
In vitro interactions improve the in vivo recall response of CD8 T cells. P14 cells were specifically activated in vitro using gp33-41 peptide in the presence of either ova323-339 (OTII) or gp61-81 (SMARTA) peptide for 24 hrs. Activated CD8 T cells were then magnetically isolated and cultured with experienced CD4 T cells for another 24 hrs. Activated CD8 T cells were again magnetically isolated and adoptively transferred into WT mice. Animals were challenged with 5×10^4^ c.f.u. of Lmgp33 i.v. at **a**) 1 day (day +1) or **b**) 30 days (day +30) after transfer. Stimulation for ICS and flow cytometry analysis was performed on spleens taken 4 days after Lmgp33 challenge. Events were gated on live CD19−Thy1.1+CD8+. **p = 0.0079. Plots are representative from one of at least two independent experiments.

In the groups challenged immediately after transfer (day +1), there was no difference in expansion of P14 cells that had or had not received help in vitro (Fig. 5a). In contrast, when mice were rested for 30 days after transfer (day +30), P14 cells expansion was significantly greater in the mice that had received in vitro “helped” P14 cells compared to mice that received “unhelped” P14 cells (Fig. 5b). Our results also suggest that this difference in expansion was not due to differences in survival of the transferred cells prior to the challenge (data not shown). In conclusion, these results suggest that memory recall of CD8 T cells is significantly improved by direct interactions with experienced CD4 T cells that are mediated by MHC-II transferred from APCs.

## Discussion

Current consensus is that CD4 T cell help is required for differentiation of CD8 T cells into memory cells that have strong proliferative potential upon secondary challenge [11], [12], [13], [14]. Dendritic cells that present both class I and class II MHC restricted antigens on the same cell are a central feature of all models for delivery of help to CD8 T cells. At the level of cellular interactions, there are two alternative models for how help is delivered. In the “licensing model” [34], CD4 T cells deliver signals that lead to differentiation of a dendritic cell into a mature stage that is subsequently able to provide adequate differentiation signals to CD8 T cells [16], [17], [35]. In contrast, the “paracrine” model invokes three-cell clusters including an antigen-bearing DC, a specific CD4 T cell, and a specific CD8 T cell, with the release of CD4 T cell derived “helper” factors in close proximity to the CD8 T cell [20], [36], [37]. Models incorporating direct antigen-specific interactions between the CD4 and CD8 T cells have generally not been contemplated, largely because CD8 T cells in mice do not synthesize the MHC-II molecules and therefore present the relevant peptides that would allow them to stimulate a CD4 T cell. In this paper, we present data that supports the plausibility of a unique model in which direct CD4∶CD8 T cell interactions can contribute to the delivery of help to CD8 T cells. In our model, this interaction is enabled by MHC-II/peptide complexes on the CD8 T cell that were obtained by trogocytosis from its activating APC.

Trogocytosis has emerged as a mechanism by which cells that do not directly transcribe and translate mRNA for specific membrane-associated proteins can nevertheless acquire those proteins from other cells. Even though the term trogocytosis was coined not long ago [38], its history begins more than 35 years ago with observations describing the acquisition of allo-antigens on the surface of activated allo-specific T cells [39]. Nearly 20 years later, transfer of cell surface molecules to CD8 T cells was shown to require signaling from either TCR or CD28 on the surface of the T cell, and at least some of the transferred molecules were not obviously directly involved in ligand interactions between the two cells, including MHC-II [29]. More recently, transfer of membrane proteins from APC—CD45.1, allo-class I MHC, and biotinylated molecules from surface-biotinylated DCs—to virus-specific TCR transgenic cells has been seen in the acute phase of the T cell response following infection of mice with viruses expressing the antigen recognized by the T cells, but no functions were ascribed to the transferred molecules [40]. Acquisition of MHC class I (MHC-I) peptide complexes by trogocytosis may provide antigenic targets for fratricide among CTL clones [30] or may simply reduce the concentration of antigen on the surface of APCs, thereby leading to a reduction in T cell stimulus [41]. It has been proposed that MHC-I molecules obtained by CD4 T cells via trogocytosis formed the basis for a novel and direct CD4∶CD8 T cell interaction that results in the provision of help for the CD8 T cells [42], but this model fails to account for signals that trigger effector functions of the CD4 T cells themselves because the CD8 T cells in this interaction do not present MHC-II/peptide complexes and therefore rely on the likelihood of encounters while TCR triggering is still maintained in CD4 T cells by endocytosed MHC-II molecules as suggested before [43].

The function of MHC-II on T cells, either expressed directly as in human T cells or acquired by trogocytosis, has been even more of a mystery. Some investigators have proposed that acquisition of MHC-II by T cells enables them to act as an APC (T-APC), resulting in amplification of CD4 T cell responses by increasing total numbers of effective APCs [44], [45]. Others have proposed that MHC-II on T cells provides either a tolerogenic signal [46] or a signal that directs the development of CD4 T cell cytotoxic function [47]. It is also possible that MHC-II molecules on T cells could serve as a ligand for regulatory CD4 T cells (T_regs_), as suggested before [48], [49]. As of today, we are the first ones to propose and demonstrate the plausibility of a model in which MHC-II acquired by CD8 T cells via the process of trogocytosis enables them to receive help from specific CD4 T cells.

The sequence in our model begins when DCs acquire antigens—either by direct infection or by phagocytosis—and present peptides from those antigens on MHC-I and MHC-II molecules. In the absence of inflammatory signals that activate the DC, CD4 T cells must first license the DC before it can activate a CD8 T cell; when inflammatory signals are present, licensing of the DC is CD4-independent. Importantly, even in the case where licensing is CD4-dependent, the signals delivered via this CD4∶DC interaction are distinct from those that are subsequently delivered via a putative direct CD4∶CD8 T cell interaction. During its interaction with a licensed DC, a responding CD8 T cell will acquire MHC-II proteins from the DC, and some of these MHC-II molecules will be loaded with peptides that are both derived from the same antigen source that provided peptides recognized by the CD8 T cell and that stimulated the CD4 T cell response. After the CD8 T cell has acquired MHC-II peptide complexes from the DC, it can then present them to antigen-specific CD4 T cells, which deliver helper signals that endow the CD8 T cell (and/or its progeny) with the ability to subsequently proliferate rapidly in a secondary recall immune response.

The events in this model of CD4 T cell help take place in a window of time that is restricted to the relatively short period in which the CD8 T cells acquire and retain the MHC-II molecules and associated peptides from the DC. In our experiments, using a relatively large number of adoptively transferred, CFSE labeled transgenic T cells, necessary to detect early events after infection with LCMV, we show that CD8 T cells acquire MHC-II molecules even before the first cell division, and begin to lose MHC-II molecules starting at about the 5th cell division, between 42–50 hours p.i.. Even in the absence of adoptive transfer, our tetramer-enrichment experiments (Fig. 1d) demonstrate that endogenous T cells that become activated also have MHC-II molecules at 2.5 days p.i.. We believe that the onset of the decline in MHC-II molecules on CD8 T cells is likely to be coincident with the clearance of antigen due to the high amount of transgenic CD8 T cells responding to LCMV and the consequent loss of antigen-specific contacts between CD8 T cells and APC. It is well-established that once recruited into a response, CD8 T cells are programmed to continue dividing for a finite number of divisions even after antigen is cleared [50]. This antigen-independent division—together with the normal turnover of MHC-II that takes place even on a non-dividing cell—will lead to partition of a limited quantity of MHC-II between daughter cells and its eventual loss from all cells in the population, much as CFSE is lost in standard turnover experiments.

The kinetics of acquisition of MHC-II peptide complexes by CD8 T cells we report here is similar to the kinetics of other key events participating in CD8 T cell memory development that have been described by others. Using the same LCMV model as we employed, it was demonstrated that the kinetics of CD25 expression on adoptively transferred transgenic CD8 T cells following infection are very similar to the kinetics of MHC-II acquisition by CD8 T cells (Fig. 1c) [51]. More importantly, it was also found that IL-2 levels in the spleen peaked between 1–3 days p.i., and though its source is still in debate, IL-2 has been involved in the programming of the secondary recall of CD8 T cells [19], [52]. Therefore, it is only during this short, critical time window that CD8 T cells possess the molecules that they need to both stimulate a CD4 T cell and to efficiently respond to IL-2 produced by putatively the same CD4 T cell. The kinetics of CD25 expression and MHC-II acquisition by CD8 T cells are also similar to the kinetics of co-accumulation of cognate CD4 and CD8 T cells around DC in draining lymph nodes that has been observed by intravital microscopy [20], [37]. While these observations have assumed a paracrine mode of delivery of IL-2 from CD4 to CD8 T cells in these CD4∶DC∶CD8 cell clusters, it has been shown that IL-2 is secreted in a synaptic rather than multidirectional fashion [53], suggesting that IL-2 delivery is more carefully targeted to a synapsis of cell∶cell contact than suggested by a standard paracrine model. Our proposal that CD4 T cells can be directly stimulated by CD8 T cells that have acquired MHC-II peptide complexes from DC provides a resolution to these apparent contradictions.

The model for delivery of CD4 T cell help to CD8 T cells that we have proposed in this paper was based upon experiments in the mouse, one of the few mammalian species that does not directly express MHC-II on activated CD8 T cells [54]. How might this model work in humans? Expression of MHC-II on activated human CD8 T cells has been demonstrated in an enormous number of studies, including direct ex vivo analyses of CD8 T cell responses to vaccinia virus and the live-attenuated Yellow Fever Virus vaccine [55]. While these studies demonstrate that MHC-II is present on antigen-specific CD8 T cells during a primary response as soon as they are detectable in the blood, we remain blind to the phenotype of these cells during priming, both because they are below the limits of detection and because they are probably sequestered in experimentally inaccessible secondary lymphoid tissues. It is possible that during priming, human CD8 T cells both express their own MHC-II and/or acquire it from APC by trogocytosis, with either enabling interactions with CD4 T cells that could provide help signals to the CD8 T cells, just as in the model presented in this paper. This model does not address the immunological function of MHC-II that is detected on T cells in the blood at the peak of the primary effector response [55] or on bulk CD8 T cells in the blood of patients during chronic immune activation such as is associated with HIV infection [56], arthritis and lupus [57]; and therefore, the function of MHC-II on CD8 T cells during the latter stages of an immune response remains a mystery.

## Materials and Methods

### Mice

Wild type (WT) C57BL6J, CIIKO, B6.Thy1.1 and OTII mice purchased from The Jackson Laboratories (Bar Harbor, ME, USA). P14 and SMARTA mice were obtained from Dr. Rafi Ahmed (Emory University, Atlanta, GA, USA). OTI mice Rag KO were obtained from Shivaprakash Gangappa (Emory University). F5 mice were obtained from Demetrius Moskophidis (Medical College of Georgia, Augusta, GA, USA), with permission from Dimitris Kioussis (NIMR, London, UK). SM1 mice were obtained from Marc K. Jenkins (University of Minnesota, Minneapolis, MN, USA). I-Ab-gfp mice were obtained from Dr. Hidde Ploegh (MIT, Cambridge, MA, USA). P14×CIIKO were generated by backcrossing into CIIKO mice more than 10 generations. F1 (I-A^b^×I-E^k^) hybrids were created by breeding AND (B10.A) mice, obtained from Dr. Brian Evavold (Emory University), with C57BL/6J from The Jackson Laboratories. All mice were maintained under specific pathogen-free conditions at the Emory Vaccine Center, Yerkes National Primate Research Center (Atlanta, GA, USA).

### Ethics Statement

Use of all mice in this study was approved by Emory University in compliance with USDA and PHS policies by the IACUC protocol #084-2008: “The role of leukocyte sequestration in the control of viral infections” from August 12^th^, 2008 until August 12^th^, 2011.

### Pathogens and infections

Age-matched sex-matched animals were injected with either 2×10^5^ plaque forming units (p.f.u.) intraperitoneally (i.p.) or 2×10^6^ pfu intravenously (i.v.) of LCMV Armstrong 53b (Arm) virus [58] or 5×10^4^ c.f.u. i.v. of rLM-gp33 [59] obtained from Dr. Rafi Ahmed (Emory University).

### Cell suspensions and flow cytometry

Single cell suspensions as described [60]. Fluorochrome-conjugated antibodies were obtained from BD Pharmingen (San Diego, CA, USA), eBiosciences (San Diego, CA, USA) and BioLegend (San Diego, CA, USA). Intracellular cytokine staining was performed following manufacturer's protocol with either 2.5 µM of peptides or anti-CD3 (145-2C11) plus anti-CD28 (37.51) both from BD Biosciences Pharmingen, for 6 hrs in the presence of Brefeldin A (1 µl/ml GolgiPlug, BD Pharmingen). Tetramers were prepared as described [61] and tetramer enrichment protocol was performed as described [62]. Live-dead discrimination was performed using an in-house developed protocol based on Alexa Fluor 430 dye (Molecular Probes, Eugene, OR, USA). Multiparameter analysis of samples was performed on LSRII flow cytometer (BD Biosciences, San Jose, CA, USA) and results were analyzed using FlowJo software (TreeStar, Ashland, OR, USA).

### Peptides

Peptides gp33-41 (KAVYNFATM), Ova257-264 (SIINFEKL), np366-374 (ASNENMDAM), gp61-80 (GLNGPDIYKGVYQFKSVEFD), Ova323-339 (ISQAVHAAHAEINEAGR), FliC427-441 (VQNRFNSAITNLGNT) were synthesized by Microchemical Facility Core (Emory University, Atlanta, GA, USA).

### Cell culture

CD8 T cells, CD4 T cells, FMS-like tyrosine kinase 3 ligand (flt3L)-DCs, B cells and splenic macrophages were purified using autoMACS system per manufacturer's protocol (Miltenyi Biotech, Auburn, CA, USA). T cells and flt3L-DCs were cultured at a 2∶1 ratio (T∶DC) in complete RPMI media [RPMI-1640 media (Life Technologies) supplemented with 10% FBS, 50 µM 2-mercaptoethanol (Sigma), 100 U/ml penicillin G, 100 µg/ml streptomycin, 10 mM HEPES buffer, 1 mM L-glutamine and 0.5 mM sodium pyruvate] at 37°C in a 5% CO_2_ incubator. Flt3L was obtained from Dr. Bob Mittler (Emory University) and flt3L-DCs were generated as described [63]. Concanavalin A was purchased from Sigma-Aldrich (St Louis, MO, USA).

### In vitro CD4 T cell differentiation

CD4 T cells (OTII, SM1 or SMARTA) were magnetically isolated and cultured with flt3L-DCs at a 5∶1 ratio overnight with their specific peptide (ova323-339, Flic427-441 or gp61-80, respectively). Activated CD4 T cells were separated by density using Lympholite M (Cedarlane, Hornby, Ontario, Canada) and incubated in complete RPMI medium supplemented with IL-2, IL-7 (10 ng/ml, eBioscience) and IL-15 (10 ng/ml, R&D Systems, Minneapolis, MN) changing media every 2 days as described before [64]. After 4 days of culture, CD4 T cells were again magnetically isolated and cultured overnight in complete RPMI media. Finally, Live CD4 T cells were depleted of dead cells using Dead Cell Discrimination kit (Miltenyi Biotech).

### Adoptive Transfer

CD8 T cells and CD4 T cells purified as described above were counted and purity was calculated by staining with antibodies specific for the Vα and Vβ TCR domains and acquisition using FACScalibur (Becton Dickinson). Purified cells were labeled with CFSE (Molecular Probes) at 2.5 µM as described before [65] and adoptively transfer as described [66].

### Real-time PCR

2×10^6^ P14 cells were adoptively transferred into naive C57BL6 mice and infected a day later with 2×10^6^ p.f.u. LMCV i.v.. At day 1.5 following LCMV infection, spleen, PLN and MLN from either uninfected or LMCV infected mice were taken, CD8 T cells magnetically enriched and stained with CD11c, CD19, Thy1.1 and CD8a. Sorted P14 from uninfected (60 k) or infected (15 k) were pelleted and resuspended in 1 ml of Tryzol (invitrogen) and stored at −80 C. RNA was extracted using Tryzol extraction protocol, treated with DNA-free DNAse (Ambion) and cDNA was made using iSuperscript (BioRad). DNA was amplified using GoTaq PCR mastermix (Promega) using primers for MHC class II (IAβ chain, [67]), CIITA (CIITA-PIII, [68]) and B-actin [69]. PCR products were run on a 2% agarose gel and image was obtained using GelDoc XR+ system (BioRad).

### Statistical Analysis

Results are expressed as mean values ± SEM and statistical significance was determined by unpaired t-test for Fig. 5 and one way ANOVA for Fig. S5 using Prism 5 (Graphpad Software, La Jolla, CA).

## Supporting Information

Figure S1 MHC class II and CD54 are present on gp33-specific CD8 T cells after LCMV infection. **a:** CD54 and MHC class II (I-Ab) staining on P14 cells in Blood, Spleen, PLN and MLN and Spleen FMO at days 0, 2, 4, 6, 8 and 15 p.i. with 2×10^5^ p.f.u. of LCMV Arm i.p. Plots are representative of triplicates from one of two independent experiments. Events gated on live CD19−Thy1.1+CD8+ singlets. **b:** Representative gating strategy for Day 2 MLN plot.
(TIFF)

Figure S2 MHC class II is present only on specific CD8 T cells after activation. **a.** MHC class II staining on adoptively transferred OTI (ova257-64 specific) or P14 (gp33-41-specific) in spleen after 32 hrs of infection with 2×10^6^ p.f.u. LCMV Arm i.v.. Each histogram represents one mouse, plots are representative from one of two independent experiments. Events were gated on live Thy1.1+CD8+ singlets. **b.** MHC class II staining on Tg CD8 T cells, P14 and F5 cells, together in culture for 24 hrs with flt3L-DCs pulsed with an irrelevant peptide (ova257-264), np366-374 (F5p), gp33-41 (P14p) or both np366-374 and gp33-41 (F5p+P14p). Plots are representative of replicates in one of two independent experiments. Events are gated on either CD8+Thy1.1+ singlets (P14 cells) or CD8+Thy1.2+ singlets (F5 cells). **c.** MHC class II staining on adoptively transferred OTI and P14 cells, together in the same mouse, detected in spleen at days 0, 1, 2, 3 and 4 after infection with 2×10^6^ p.f.u. LCMV Arm i.v. Plots are representatives of duplicates in one of two experiments.
(TIFF)

Figure S3 MHC class II is present on blasting endogenous CD8 T cells responding to LCMV infection. **a.** MHC-II (I-A^b^-gfp) vs CD25 staining on activated D(b)/LCMV.gp33-41 (KAVYNFATM) tetramer enriched CD8 T cells 2.5 days p.i. with 2×10^6^ of LCMV Arm i.v.. Events were gated on live CD19−CD11b−CD4−CD8+ KAVYNFATM-tetramer+ singlets. Plot is representative of triplicates from one of two independent experiments. **b.** SSC of CD25+MHCII+ KAVYNFATM-tet+ enriched endogenous CD8 T cells (blue) vs CD25-MHCII- KAVYNFATM-tet+ enriched endogenous CD8 T cells (red) overlayed to the bulk population of CD19−CD11b−CD4−CD8+ T cells singlets (solid grey). **c.** MHC class II (I-Ab-gfp) vs CD25 staining on K(b).Ovalbumin257-64 (SIINFEKL) tetramer enriched CD8 T cells 2.5 days post-infection with 2×10^6^ of LCMV Arm i.v. One graph per mouse. Events were gated on live CD19−CD11b−CD4−CD8+ SIINFEKL-tet+ singlets.
(TIFF)

Figure S4 MHC-II is not present on CD8 T cells activated by CIIKO DCs. **a.** PCR products with their approx. band size (bp, right) was obtained using primers to amplify MHC class II, CIITA and β-actin on cDNA made by RT-PCR from magnetically isolated CIIKO DCs and WT DCs as well as from FACS sorted uninfected (Uninf) and infected (LCMV) P14 cells. **b.** FMO control and MHC-II staining vs CFSE dilution on CD8 T cells (P14) in CIIKO mice at 0, 36 and 62 hrs after infection with 2×10^6^ p.f.u. LCMV Arm i.v.. Plots are representative of triplicates. Events were gated on live CD19−CD11c−Thy1.1+CD8+ singlets. **c.** MHC-II and CD25 staining on Tg CD8 T cells (P14 or P14×CIIKO) cultured in vitro for 24 hrs with cognate peptide using flt3L-DCs from either CIIKO or WT mice. Events were gated on live CD19−CD8+ singlets.
(TIFF)

Figure S5 CD11c+ APCs transfer most of MHC-II observed on activated CD8 T cells. **a.** CD11c vs CD11b define magnetically enriched APC populations (B220+, CD11b+ or CD11c+) cultured in vitro with CD8 T cells. Events were gated on live singlets. **b.** Comparable amounts of MHC Class II on magnetically enriched APC populations (B220+, CD11b+ or CD11c+) cultured in vitro with CD8 T cells. MFI values of I-Ab-APC, calculated on events gated respectively on CD19+, CD11b+ or CD11c+ live singlets in **a**. **c.** Tg CD8 T cells (P14) were cultured in vitro with control (ova257-64, solid histogram) or cognate (gp33-41, empty histogram) peptide for 24 hrs using different magnetically enriched APCs (B220+, CD11b+ and CD11c+). Events were gated on live CD19− Thy1.1+CD8+ singlets. **d.** MFI of MHC Class II (I-A^b^) on activated CD8 T cells portrayed in **c**. Events were gated on live CD19− Thy1.1+CD8+ singlets. *p = 0.0157.
(TIFF)

Figure S6 CD4 T cell stimulation with activated CD8 T cells is not due to DC contamination. **a.** Little DC contamination can be detected on purified activated CD8 T cells. CD11c vs CD19 on ungated total cells from magnetically purified CD8 T cells after 24 hrs of in vitro activation with flt3L-DCs. CD8 T (P14) cells were primed in the presence of gp33-41 peptide either with ova323-339 (OTIIp) or with gp61-80 (SMARTAp). flt3L-DCs cultured for 24 hrs in the presence of SMARTA peptide were added to activated CD8 T cells loaded with OTII peptide to control for possible DC contamination. Events were gated on live singlets. **b.** Residual DC contamination after magnetic isolation of activated CD8 T cells is not responsible for CD4 T cell stimulation. TNFα vs IL2 expression detected using intracellular cytokine staining by flow cytometry. Events were gated on live CD19−Thy1.2+CD4+ singlets. Direct CD4 T cell stimulation by activated CD8 T cells isolated as described in **a**. Contaminating DCs are added to activated CD8 T cell before magnetic isolation. **c.** CD4 T cell responses are mainly caused by peptides presented by CD8 T cells. TNFα vs IL2 expression detected using intracellular cytokine staining by flow cytometry. Events were gated on live CD19−Thy1.2+CD4+ singlets. Ratio of flt3L-Dcs to both CD8 T cells and CD4 T cells (1×10^5^ cells per well, 1∶1 CD8 to CD4 T cell ratio).
(TIFF)
