# Supplementary figures and images for: CD8 T Cell Memory Recall Is Enhanced by Novel Direct Interactions with CD4 T Cells Enabled by MHC Class II Transferred from APCs
Source: PLoS One. 2013 Feb 18;8(2):e56999. doi: 10.1371/journal.pone.0056999 (PMC3575485; doi:10.1371/journal.pone.0056999)

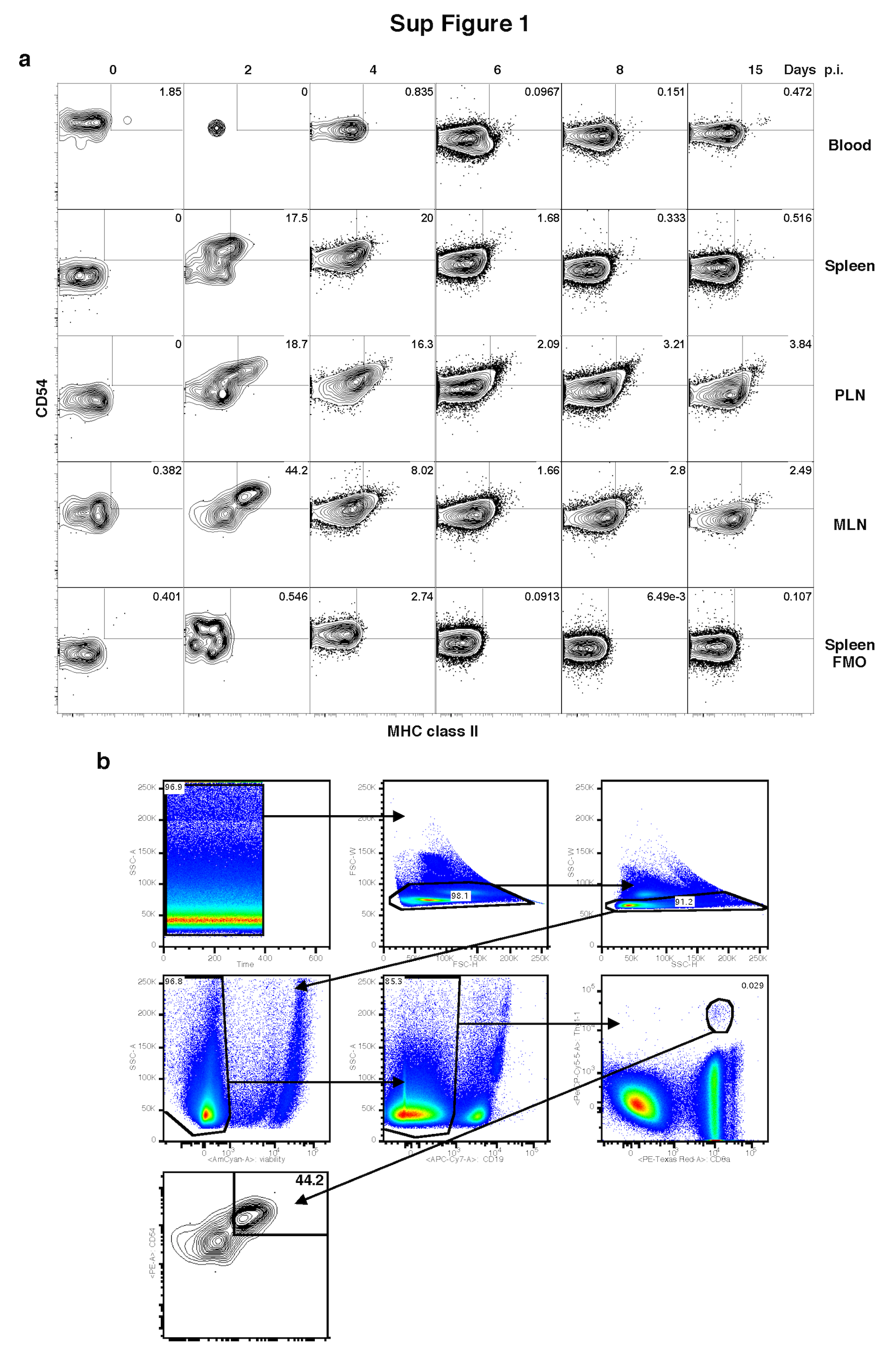

Supplement: Figure S1 — MHC class II and CD54 are present on gp33-specific CD8 T cells after LCMV infection. a: CD54 and MHC class II (I-Ab) staining on P14 cells in Blood, Spleen, PLN and MLN and Spleen FMO at days 0, 2, 4, 6, 8 and 15 p.i. with 2×10^5^ p.f.u. of LCMV Arm i.p. Plots are representative of triplicates from one of two independent experiments. Events gated on live CD19−Thy1.1+CD8+ singlets. b: Representative gating strategy for Day 2 MLN plot. (TIFF) [file pone.0056999.s001.tiff]

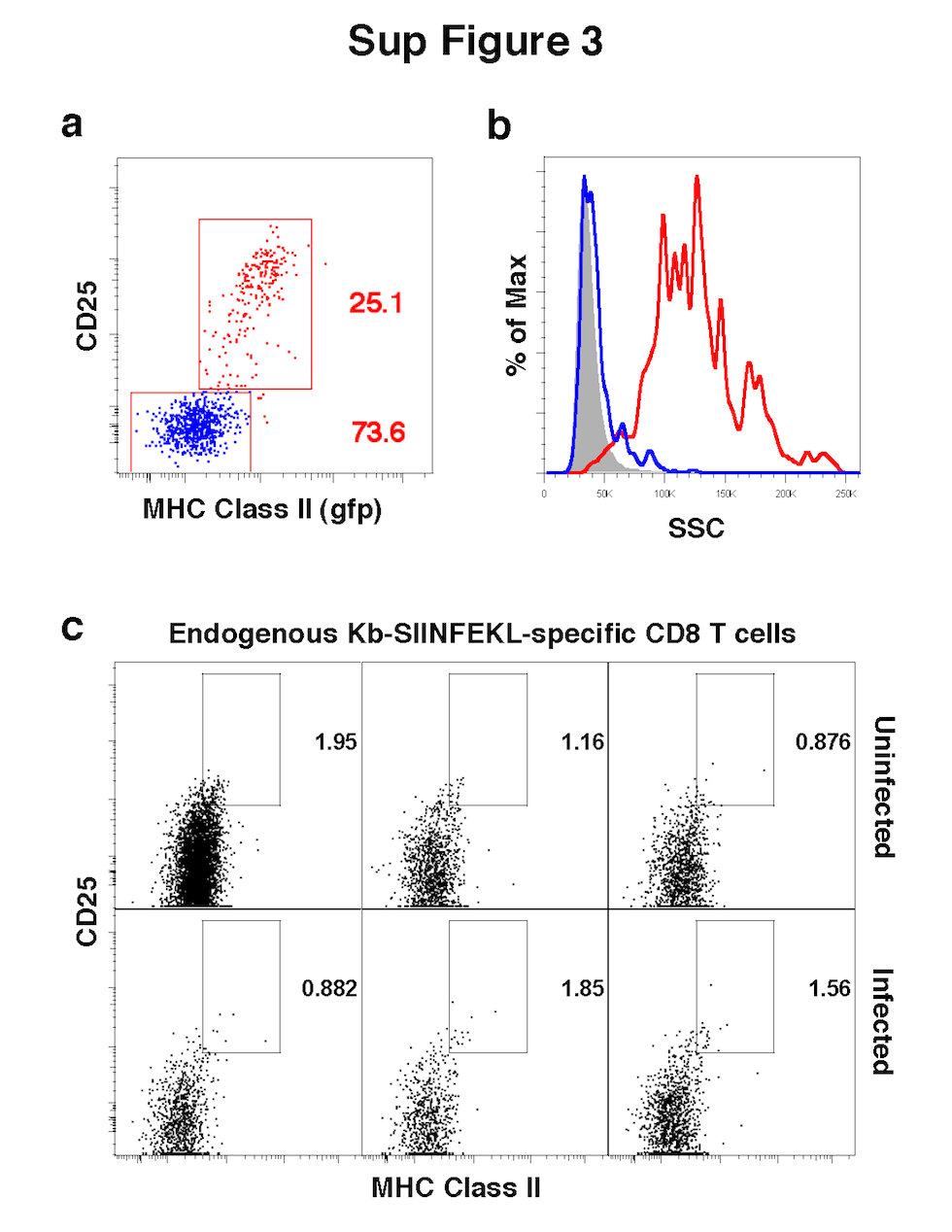

Supplement: Figure S3 — MHC class II is present on blasting endogenous CD8 T cells responding to LCMV infection. a. MHC-II (I-Ab-gfp) vs CD25 staining on activated D(b)/LCMV.gp33-41 (KAVYNFATM) tetramer enriched CD8 T cells 2.5 days p.i. with 2×10^6^ of LCMV Arm i.v.. Events were gated on live CD19−CD11b−CD4−CD8+ KAVYNFATM-tetramer+ singlets. Plot is representative of triplicates from one of two independent experiments. b. SSC of CD25+MHCII+ KAVYNFATM-tet+ enriched endogenous CD8 T cells (blue) vs CD25-MHCII- KAVYNFATM-tet+ enriched endogenous CD8 T cells (red) overlayed to the bulk population of CD19−CD11b−CD4−CD8+ T cells singlets (solid grey). c. MHC class II (I-Ab-gfp) vs CD25 staining on K(b).Ovalbumin257-64 (SIINFEKL) tetramer enriched CD8 T cells 2.5 days post-infection with 2×10^6^ of LCMV Arm i.v. One graph per mouse. Events were gated on live CD19−CD11b−CD4−CD8+ SIINFEKL-tet+ singlets. (TIFF) [file pone.0056999.s003.tiff]

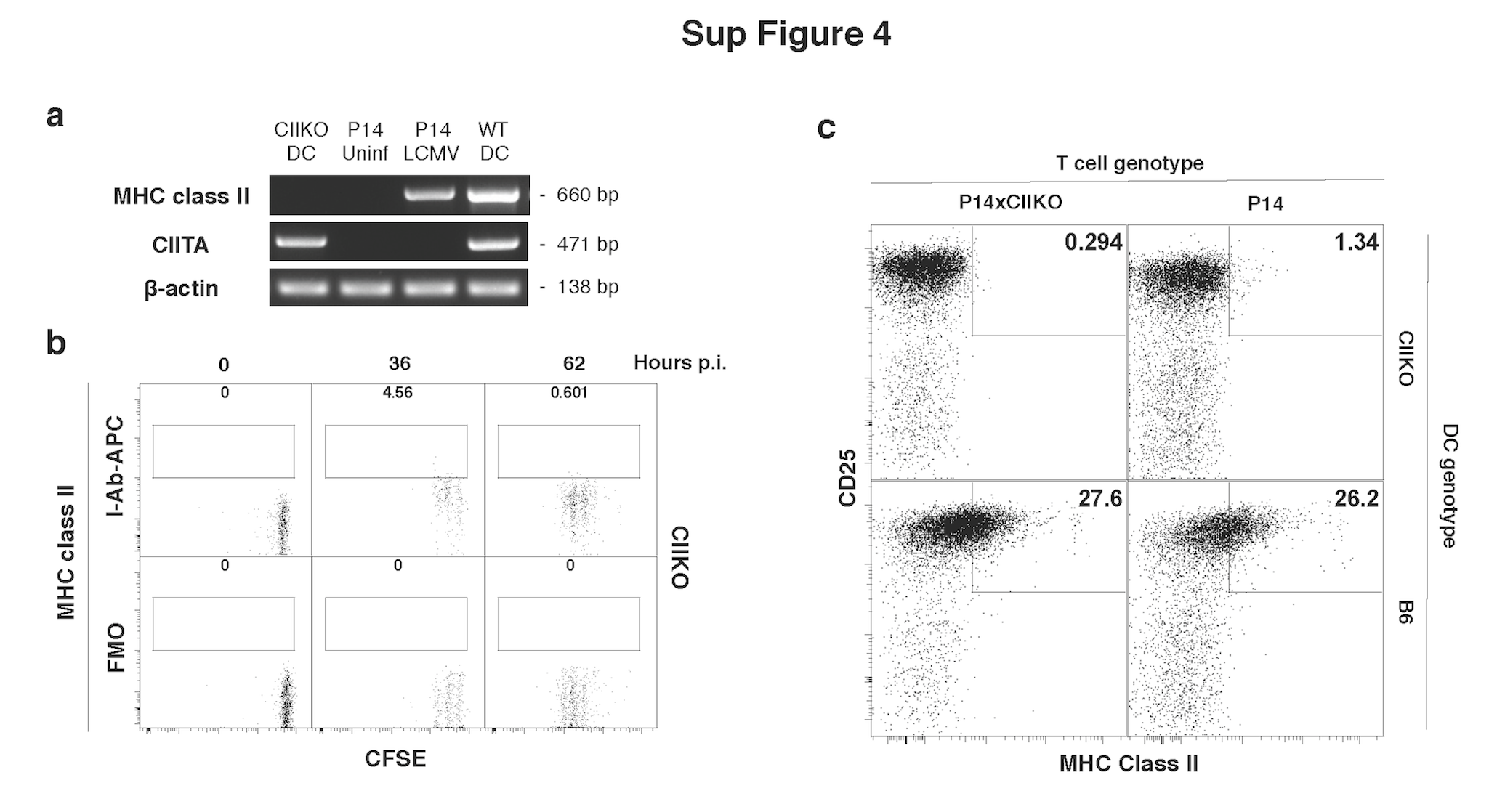

Supplement: Figure S4 — MHC-II is not present on CD8 T cells activated by CIIKO DCs. a. PCR products with their approx. band size (bp, right) was obtained using primers to amplify MHC class II, CIITA and β-actin on cDNA made by RT-PCR from magnetically isolated CIIKO DCs and WT DCs as well as from FACS sorted uninfected (Uninf) and infected (LCMV) P14 cells. b. FMO control and MHC-II staining vs CFSE dilution on CD8 T cells (P14) in CIIKO mice at 0, 36 and 62 hrs after infection with 2×10^6^ p.f.u. LCMV Arm i.v.. Plots are representative of triplicates. Events were gated on live CD19−CD11c−Thy1.1+CD8+ singlets. c. MHC-II and CD25 staining on Tg CD8 T cells (P14 or P14×CIIKO) cultured in vitro for 24 hrs with cognate peptide using flt3L-DCs from either CIIKO or WT mice. Events were gated on live CD19−CD8+ singlets. (TIFF) [file pone.0056999.s004.tiff]

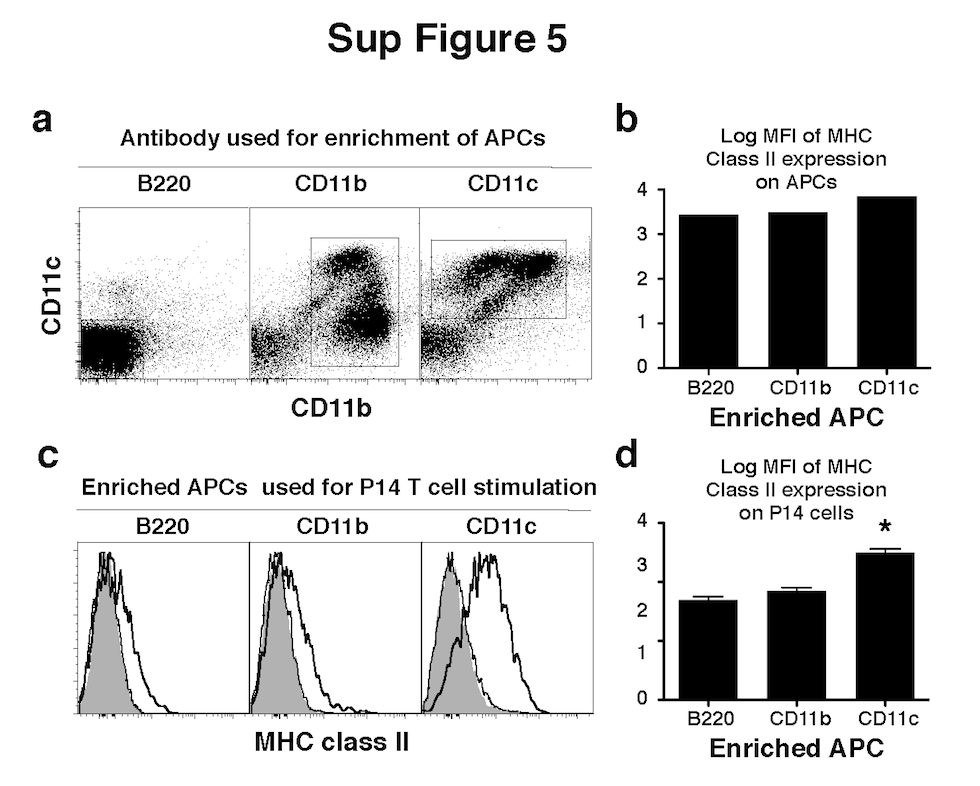

Supplement: Figure S5 — CD11c+ APCs transfer most of MHC-II observed on activated CD8 T cells. a. CD11c vs CD11b define magnetically enriched APC populations (B220+, CD11b+ or CD11c+) cultured in vitro with CD8 T cells. Events were gated on live singlets. b. Comparable amounts of MHC Class II on magnetically enriched APC populations (B220+, CD11b+ or CD11c+) cultured in vitro with CD8 T cells. MFI values of I-Ab-APC, calculated on events gated respectively on CD19+, CD11b+ or CD11c+ live singlets in a. c. Tg CD8 T cells (P14) were cultured in vitro with control (ova257-64, solid histogram) or cognate (gp33-41, empty histogram) peptide for 24 hrs using different magnetically enriched APCs (B220+, CD11b+ and CD11c+). Events were gated on live CD19− Thy1.1+CD8+ singlets. d. MFI of MHC Class II (I-Ab) on activated CD8 T cells portrayed in c. Events were gated on live CD19− Thy1.1+CD8+ singlets. *p = 0.0157. (TIFF) [file pone.0056999.s005.tiff]

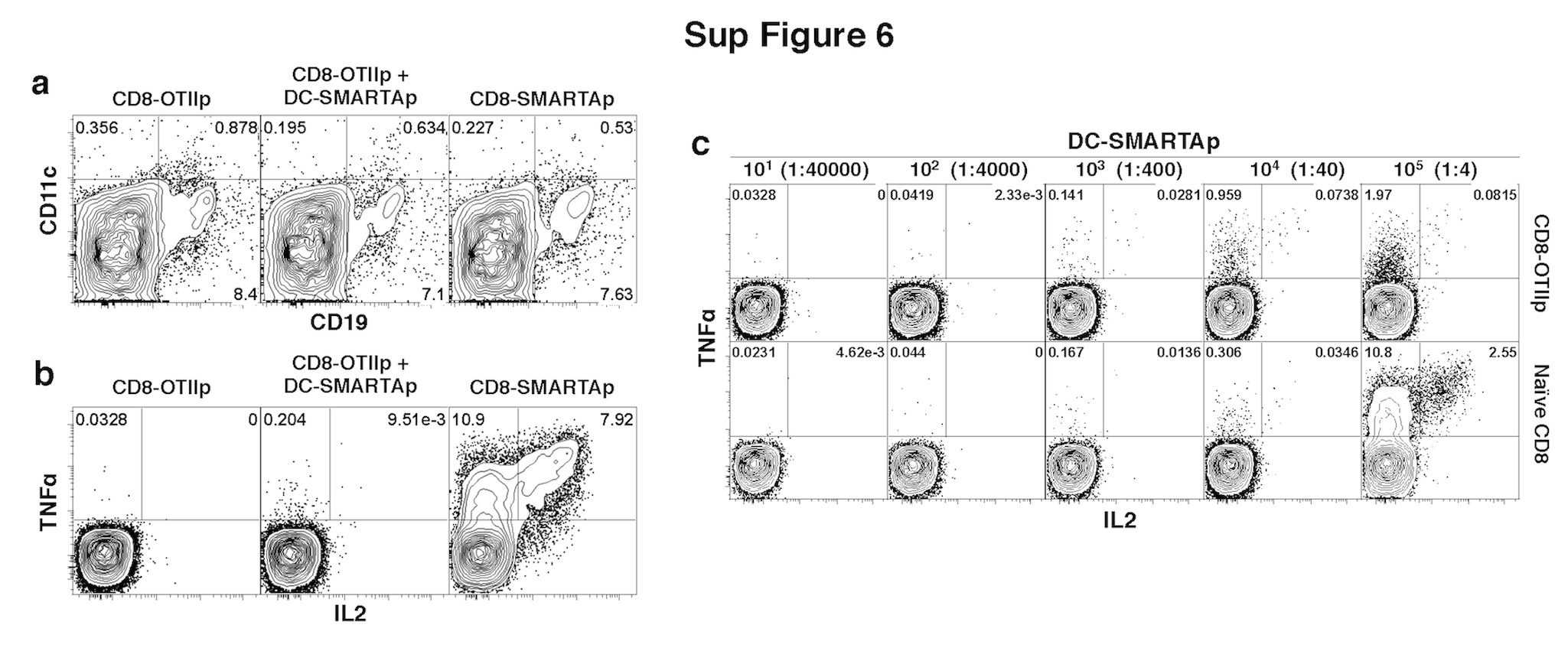

Supplement: Figure S6 — CD4 T cell stimulation with activated CD8 T cells is not due to DC contamination. a. Little DC contamination can be detected on purified activated CD8 T cells. CD11c vs CD19 on ungated total cells from magnetically purified CD8 T cells after 24 hrs of in vitro activation with flt3L-DCs. CD8 T (P14) cells were primed in the presence of gp33-41 peptide either with ova323-339 (OTIIp) or with gp61-80 (SMARTAp). flt3L-DCs cultured for 24 hrs in the presence of SMARTA peptide were added to activated CD8 T cells loaded with OTII peptide to control for possible DC contamination. Events were gated on live singlets. b. Residual DC contamination after magnetic isolation of activated CD8 T cells is not responsible for CD4 T cell stimulation. TNFα vs IL2 expression detected using intracellular cytokine staining by flow cytometry. Events were gated on live CD19−Thy1.2+CD4+ singlets. Direct CD4 T cell stimulation by activated CD8 T cells isolated as described in a. Contaminating DCs are added to activated CD8 T cell before magnetic isolation. c. CD4 T cell responses are mainly caused by peptides presented by CD8 T cells. TNFα vs IL2 expression detected using intracellular cytokine staining by flow cytometry. Events were gated on live CD19−Thy1.2+CD4+ singlets. Ratio of flt3L-Dcs to both CD8 T cells and CD4 T cells (1×10^5^ cells per well, 1∶1 CD8 to CD4 T cell ratio). (TIFF) [file pone.0056999.s006.tiff]
